# The extracellular matrix and focal adhesion kinase signaling regulate cancer stem cell function in pancreatic ductal adenocarcinoma

**DOI:** 10.1371/journal.pone.0180181

**Published:** 2017-07-10

**Authors:** Asma Begum, Theodore Ewachiw, Clinton Jung, Ally Huang, K. Jessica Norberg, Luigi Marchionni, Ross McMillan, Vesselin Penchev, N. V. Rajeshkumar, Anirban Maitra, Laura Wood, Chenguang Wang, Christopher Wolfgang, Ana DeJesus-Acosta, Daniel Laheru, Irina M. Shapiro, Mahesh Padval, Jonathan A. Pachter, David T. Weaver, Zeshaan A. Rasheed, William Matsui

**Affiliations:** 1 Departments of Oncology, The Sidney Kimmel Comprehensive Cancer Center, Johns Hopkins University School of Medicine, Baltimore, Maryland, United States of America; 2 Department of Pathology, University of Texas M.D. Anderson Cancer Center, Houston, Texas, United States of America; 3 Department of Pathology, Johns Hopkins University School of Medicine, Baltimore, Maryland, United States of America; 4 Department of Surgery, Johns Hopkins University School of Medicine, Baltimore, Maryland, United States of America; 5 Verastem, Inc., Needham, Massachusetts, United States of America; University of Nebraska Medical Center, UNITED STATES

## Abstract

Cancer stem cells (CSCs) play an important role in the clonogenic growth and metastasis of pancreatic ductal adenocarcinoma (PDAC). A hallmark of PDAC is the desmoplastic reaction, but the impact of the tumor microenvironment (TME) on CSCs is unknown. In order to better understand the mechanisms, we examined the impact of extracellular matrix (ECM) proteins on PDAC CSCs. We quantified the effect of ECM proteins, β1-integrin, and focal adhesion kinase (FAK) on clonogenic PDAC growth and migration *in vitro* and tumor initiation, growth, and metastasis *in vivo* in nude mice using shRNA and overexpression constructs as well as small molecule FAK inhibitors. Type I collagen increased PDAC tumor initiating potential, self-renewal, and the frequency of CSCs through the activation of FAK. FAK overexpression increased tumor initiation, whereas a dominant negative FAK mutant or FAK kinase inhibitors reduced clonogenic PDAC growth *in vitro* and *in vivo*. Moreover, the FAK inhibitor VS-4718 extended the anti-tumor response to gemcitabine and nab-paclitaxel in patient-derived PDAC xenografts, and the loss of FAK expression limited metastatic dissemination of orthotopic xenografts. Type I collagen enhances PDAC CSCs, and both kinase-dependent and independent activities of FAK impact PDAC tumor initiation, self-renewal, and metastasis. The anti-tumor impact of FAK inhibitors in combination with standard chemotherapy support the clinical testing of this combination.

## Introduction

Pancreatic ductal adenocarcinoma (PDAC) leads to an estimated 40,000 deaths annually and is the fourth leading cause of cancer related deaths in the United States [1]. Despite the approval of new agents, survival rates remain low, and fewer than 5% of patients are alive 5 years after diagnosis [2]. Disease recurrence following surgical resection and metastatic dissemination drives poor outcomes, and tumor initiating cells (TICs) or cancer stem cells (CSCs) have been implicated in both processes [2–7]. The factors regulating CSCs are not fully understood, but the stem cell niche composed of non-stem cells, extracellular matrix (ECM) proteins, and specific signaling molecules maintains the functional properties of normal self-renewing stem cells [8–10]. In PDAC, the tumor microenvironment (TME) is dramatically altered by the densely fibrotic desmoplastic reaction [11–14], and several ECM proteins, including collagen, fibronectin, and laminin, are highly overexpressed [14]. Each of these ECM molecules has been found to promote the growth and invasion of PDAC cells [15–19], and we examined their impact on CSCs.

Type I collagen is the most abundant ECM protein in the TME and is associated with poor survival in PDAC [13]. In PDAC, type I collagen promotes cancer cell invasion, migration and drug resistance [20–26]. Furthermore, type I collagen can promote an immature cellular phenotype similar to CSCs in colorectal cancer [27]. The major cellular receptors for ECM proteins are integrins composed of α and β subunits. Type I collagen binding through β1 integrin, in combination with multiple α subunits, leads to the activation of several intracellular signaling pathways, including focal adhesion kinase (FAK) [28, 29]. FAK (*FAK1*) is a multifunctional non-receptor protein tyrosine kinase, encoded by the PTK2 (protein tyrosine kinase 2) gene located at chromosome 8q24.3. FAK is required for normal embryonic development [30, 31], overexpressed in thyroid, colorectal, prostate, and ovarian carcinomas, and associated with disease progression and poor outcomes [32]. Recent studies have also demonstrated that FAK regulates CSC properties in breast, gastrointestinal, and squamous cell carcinoma [33–35]. In PDAC, FAK expression has been associated with survival, metastasis, and drug resistance [36–41], but its impact on pancreatic CSCs is not well understood.

We examined the effect of specific ECM proteins on the clonogenic growth of PDAC cells and found that type I collagen enhanced tumor initiating potential and self-renewal. We also found that these effects are dependent on β1 integrin expression and FAK activation. We previously demonstrated that pancreatic CSCs express increased levels of aldehyde dehydrogenase (ALDH) and are associated with metastatic disease [6]. Type I collagen also increased the frequency of ALDH^+^ PDAC cells, and β1 integrin-FAK expression was enriched in ALDH^+^ CSCs. Furthermore, β1 integrin and FAK activation were required for pancreatic CSC self-renewal and migration, and FAK inhibition abrogates clonogenic PDAC growth *in vitro* and *in vivo*. Therefore, type I collagen within the PDAC desmoplastic reaction enhances CSCs and promotes tumor initiation, self-renewal, and metastasis through β1 integrin-FAK signaling.

## Results

### Type I collagen- β1 integrin signaling enhances the clonogenic growth of pancreatic cancer cells

**Fig 1:**
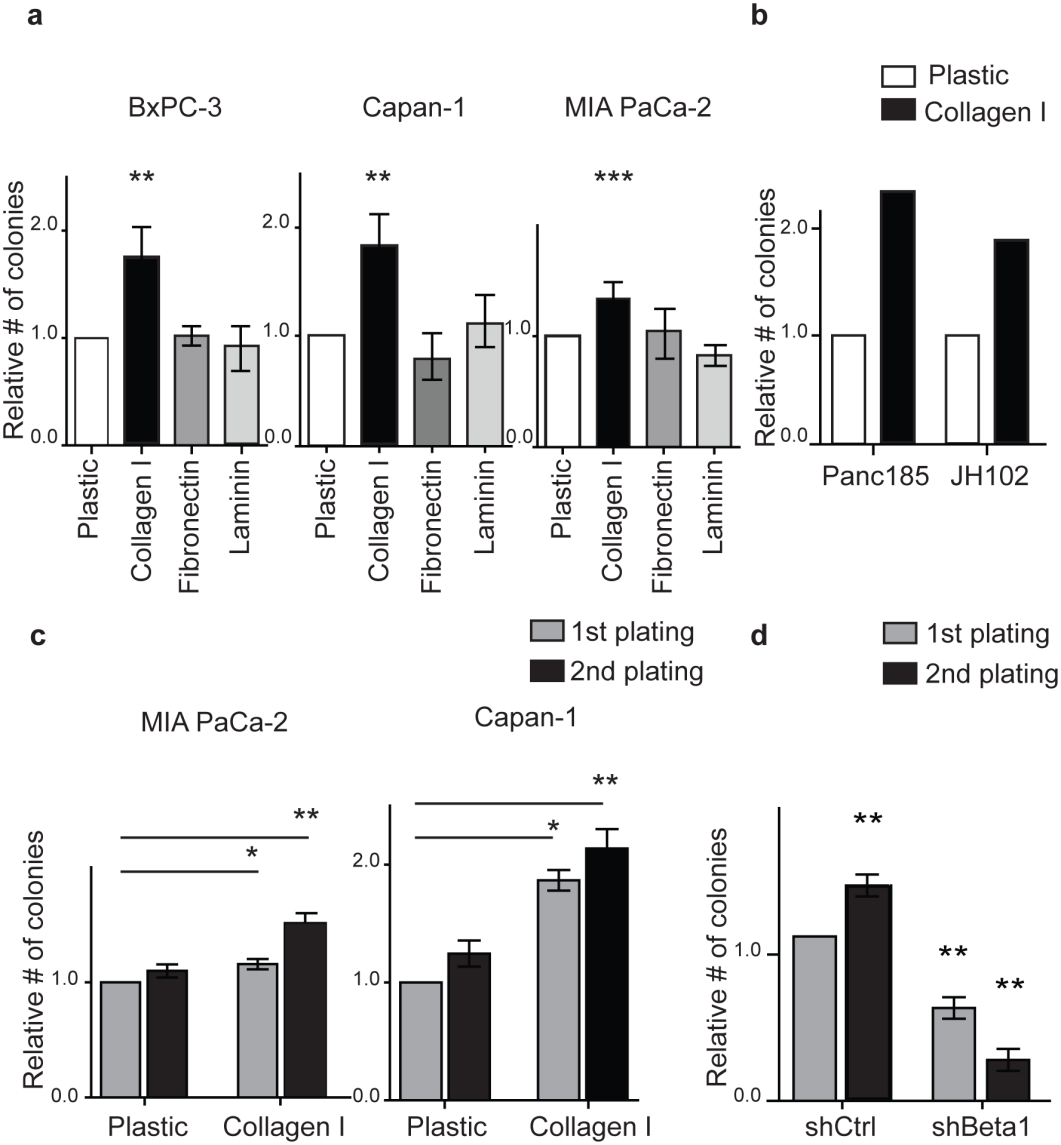
Type I collagen- β1 integrin signaling enhances the clonogenic growth of PDAC cells. (**a**) Colony formation by BxPC-3, Capan-1 and MIA PaCa-2 cells following growth on type I collagen, fibronectin, or laminin for 96 hours. Data represent the mean ± SD (*n* = 4) compared to control cell growth on plastic; **P < 0.001; ***P < 0.0001 by ANOVA. (**b**) Colony formation assay by cells from 2 distinct patient derived xenografts cells cultured on plastic or type I collagen for 96 hours. (**c**) Secondary colony formation by MIA PaCa-2, and Capan-1 cells. Data represent the mean ± SD (*n* = 4) compared to control; *P < 0.05, **P < 0.001. (**d**) Primary and secondary colony formation by MIA PaCa2 cells expressing a scrambled control (shCtrl) or β1 integrin (shBeta1) shRNA after culture on type I collagen for 96 hours. Data represent mean ± SD (*n* = 4). **P < 0.001.

Specific ECM proteins are abundantly and aberrantly expressed in the desmoplastic TME of both primary and metastatic PDAC tumors [13, 14]. Several of these, including type I collagen, fibronectin and laminin, promote PDAC cell survival, proliferation, and migration [15–19], but their impact on clonogenic tumor growth is not fully understood. We cultured BxPC-3, Capan-1, and MIA PaCa-2 cells on each of these ECM proteins for 96 hours, and then quantified tumor cell colony formation in methylcellulose. Compared to control cells cultured on plastic alone, cells grown on type I collagen, but not fibronectin or laminin, formed significantly more colonies in all 3 cell lines (Fig 1a; 1.4–1.7 fold). Similarly, type I collagen also enhanced the clonogenic potential of PDAC cells isolated from low passage xenografts derived from primary surgical specimens by 1.8–2.3 fold (Fig 1b). To examine the impact of type I collagen on the self renewal and maintenance of clonogenic PDAC growth, we harvested tumor colonies and replated single cells in methylcellulose. Although cells were not further exposed to type I collagen, secondary colony formation by MIA PaCa-2 and Capan-1 cells was significantly increased by 1.6–2.2 fold (Fig 1c). Therefore, type I collagen increases the clonogenic growth and self-renewal of PDAC cells.

Integrins are major cellular receptors for ECM proteins and heterodimers composed of α and β subunits [42, 43]. Type I collagen binding involves several α integrin subunits but only a single β subunit, β1-integrin. β1-integrin is highly expressed by normal stem cells in the hematopoietic and central nervous systems [44, 45] and promotes PDAC cell proliferation and migration [46]. To determine whether β1-integrin mediates the effects of type I collagen on clonogenic PDAC growth, we knocked down its expression in PDAC cells by shRNA (S1a Fig). Compared to a control scrambled shRNA construct, the loss of β1-integrin significantly inhibited primary and secondary colony formation by MIA PaCa-2 (Fig 1d) and Capan-1cells cultured on type I collagen by >2 fold (S1b Fig). The impact of β1-integrin loss was not a consequence of cell death as apoptosis was not increased (S1c Fig). Thus, β1-integrin mediates the effects of type I collagen on PDAC tumor initiation and self-renewal.

### FAK overexpression increases PDAC clonogenic growth and tumor initiating capacity

**Fig 2:**
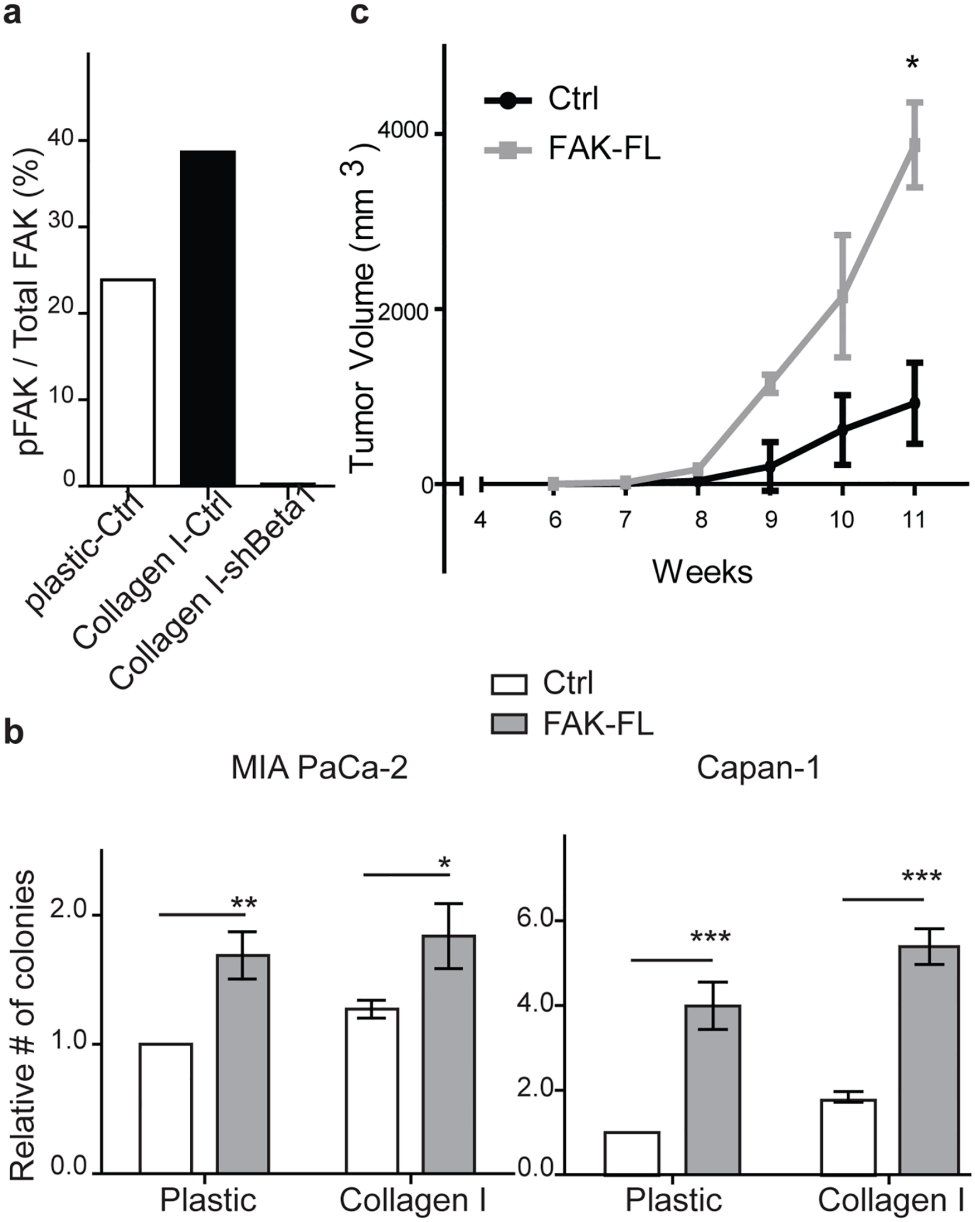
FAK overexpression increases PDAC clonogenic growth and tumor initiating capacity. (**a**) Ratio of phospho-FAK to total FAK expression by MIA PaCa2 cells expressing a scrambled control (shCtrl) or β1 integrin (shBeta1) shRNA and cultured on plastic or type I collagen for 96 hours. (**b**) Colony formation by MIA PaCa-2 and Capan-1 cells overexpressing FAK-FL following growth on plastic or type I collagen for 96 hours. Data represent mean ± SD (*n* = 4) of control versus FAK-FL; *P < 0.05; **P < 0.001; ***P < 0.0001. (**c**) Tumor growth of MIA PaCa2 cells overexpressing FAK-FL following subcutaneously injection in to NSG mice. *P = 0.04 compared to control (vector).

Integrin signaling involves the activation of several intracellular pathways, including Integrin Linked Kinase, SRC family kinases, and Focal Adhesion Kinase (FAK). FAK in particular has been implicated in regulating CSCs in squamous cell skin and breast carcinoma [34, 36]. FAK activation involves the phosphorylation of tyrosine-397 (pFAK-Y397), and we found that pFAK-Y397 expression increased when PDAC cells were cultured on type I collagen (Fig 2a). Furthermore, β1-integrin expression was required for FAK activation as β1-integrin knock down led to the loss of pFAK-Y397 (Fig 2a).

To investigate the role of FAK signaling in PDAC self-renewal and tumor-initiation potential, we overexpressed full length wild-type FAK (FAK-FL) in MIA PaCa-2 and Capan-1 cells. FAK-FL overexpression increased the level of activated pFAK-Y397 (S2a Fig) and significantly enhanced tumor colony formation by 1.6–5 fold (Fig 2b) compared to cells transduced with an empty vector construct. Furthermore, clonogenic growth was similarly increased for cells cultured on either plastic or type I collagen suggesting that FAK mediates the response to type I collagen. We also studied the impact of FAK on *in vivo* tumor formation and found that the tumor-initiating cell (TIC) frequency of FAK-FL overexpressing MIA PaCa-2 cells was significantly higher than control cells (1/28 vs. 1/314; Table 1). Furthermore, FAK-FL overexpression significantly increased the growth rate of established tumors (Fig 2c). Therefore, FAK activation enhances the tumor initiating potential and growth of PDAC cells.

**Table 1:** FAK regulates tumor initiating cell (TIC) frequency in PDAC.

# of cells injected	Tumor incidence		
	Ctrl (vec)	FAK-FL	FAK-Y397F
10,000	2/2	2/2	4/4
1,000	4/4	4/4	1/4
500	1/4	4/4	1/4
100	6/10	6/6	1/10
10	--	2/8	--
TIC frequency	1 in 314	1 in 28	1 in 986
TIC 95% CI	1/656–1/151	1/69–1/11	1/2393–1/406
*p*- Value		*p* < 0.001	*p* = 0.038

Frequency of tumor initiation by MIA PaCa2 cells overexpressing FAK-FL or FAK-Y397F as assessed by limiting dilution assay. Control versus FAK-FL, *P* < 0.001; control versus FAK-Y397F, *P* = 0.038.

### Type I collagen-β1 integrin-FAK signaling impacts ALDH^+^ PDAC CSCs

**Fig 3:**
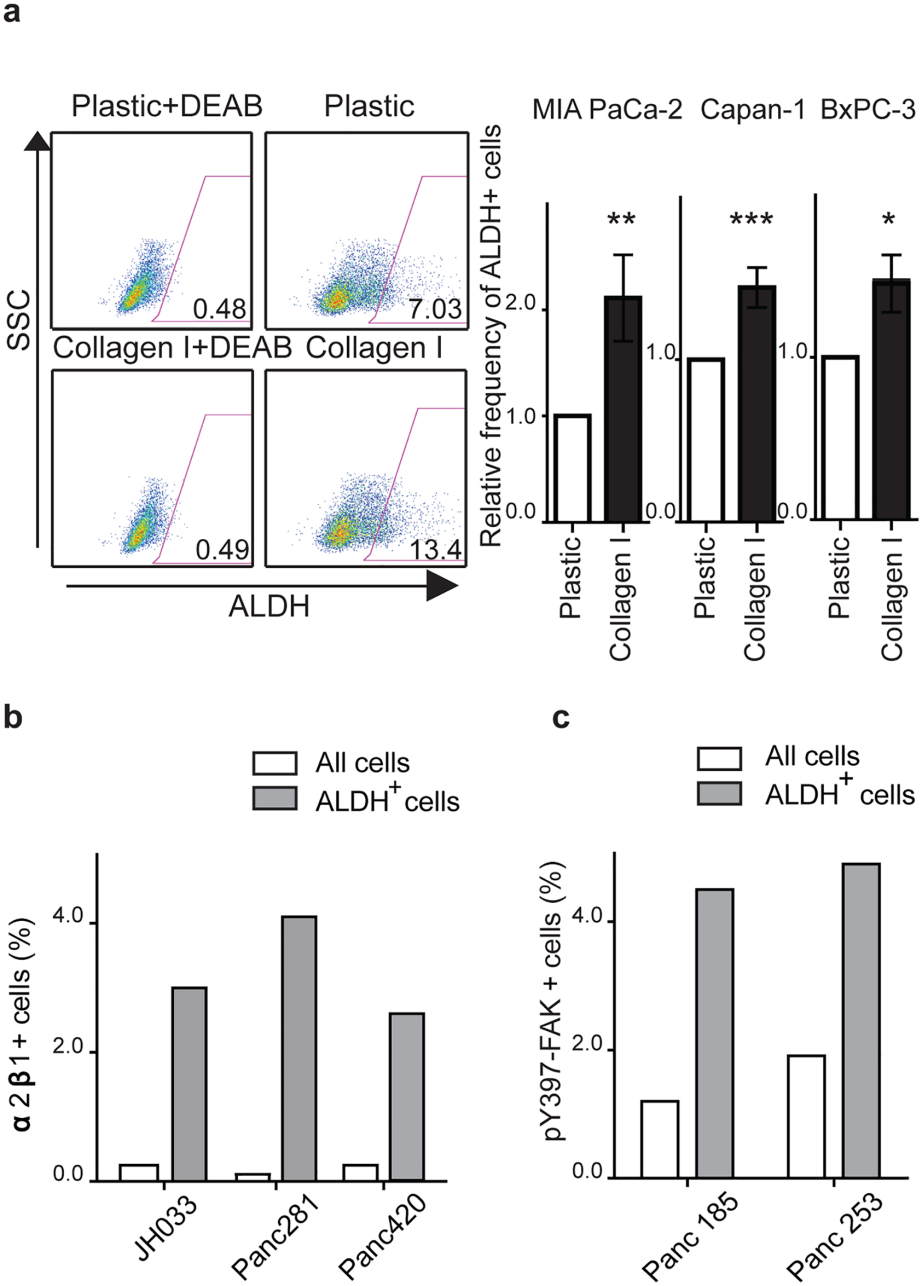
Type I collagen-β1 integrin-FAK signaling impacts ALDH^+^ PDAC CSCs. (**a**) Frequency of ALDH^+^ MIA PaCa-2, Capan-1, and BxPC-3 cells following culture on plastic or type I collagen for 96 hours. Data represent mean ± SD (*n* = 3) compared to cell growth on plastic; *P < 0.05, **P < 0.001, ***P < 0.0001. (**b**) Frequency of α2β1^+^ cells within ALDH^+^ or all tumor cells from 3 distinct patient derived xenografts. (**c**) Frequency of phospho-FAK^+^ cells within ALDH^+^ or all tumor cells from 2 distinct patient derived xenografts as determined by flow cytometry.

We previously demonstrated that ALDH^+^ PDAC CSCs are highly clonogenic and associated with inferior overall survival in patients with resected PDAC [6]. We also found that ALDH^+^ cells are enriched at the interface between tumor cells and the desmosplastic reaction, suggesting that CSCs specifically interact with the TME [6]. To determine the impact of type I collagen on PDAC CSCs, we cultured BxPC-3, Capan-1, and MIA PaCa-2 cells on type I collagen and quantified ALDH^+^ CSCs by flow cytometry. Compared to cells cultured on plastic, the frequency of ALDH^+^ cells significantly increased in all three cell lines by 1.3–2.2 fold following growth on type I collagen (Fig 3a). Although several α subunits can bind to type I collagen in association with β1 integrin, α2β1 has been found to enhance the proliferation and migration of PDAC cells [19]. Furthermore, α2β1-integrin modulates cell fate decisions in mesenchymal stem cells and is expressed by CSCs in prostate cancer [47]. Similarly, in MIA PaCa-2 cells, surface α2β1-integrin was expressed by almost half (47.9%) of ALDH^+^ CSCs but only a minority (2.2%) of bulk tumor cells (S3a Fig). In patient-derived low-passage xenografts, α2β1-integrin was expressed by 0.2–0.4% of bulk tumor cells and 2.8–4.3% of ALDH^+^ cells, a 12–37 fold increase by CSCs (Fig 3b). We also examined the expression of activated FAK and found that 30–100% of ALDH^+^ cells within PDAC cell lines expressed pY397-FAK compared to 7–44% of bulk tumor cells (S3b Fig). Similarly, in low-passage xenografts pY397-FAK was expressed by 4–5% of ALDH^+^ CSCs compared to 1.2–2% of bulk tumor cells (Fig 3c). The frequency of ALDH^+^ cells was impacted by β1-integrin-FAK signaling as FAK-FL overexpression increased CSCs by >1.3 fold whereas β1-integrin knock-down led to a significant decrease in ALDH^+^ MIA PaCa-2 cells by 5-fold (S3c and S3d Fig). Therefore, type I collagen-β1-integrin-FAK signaling impacts ALDH^+^ PDAC CSCs.

### FAK kinase-inhibition decrease clonogenic PDAC growth *in vitro* and inhibits tumor growth *in vivo*

**Fig 4:**
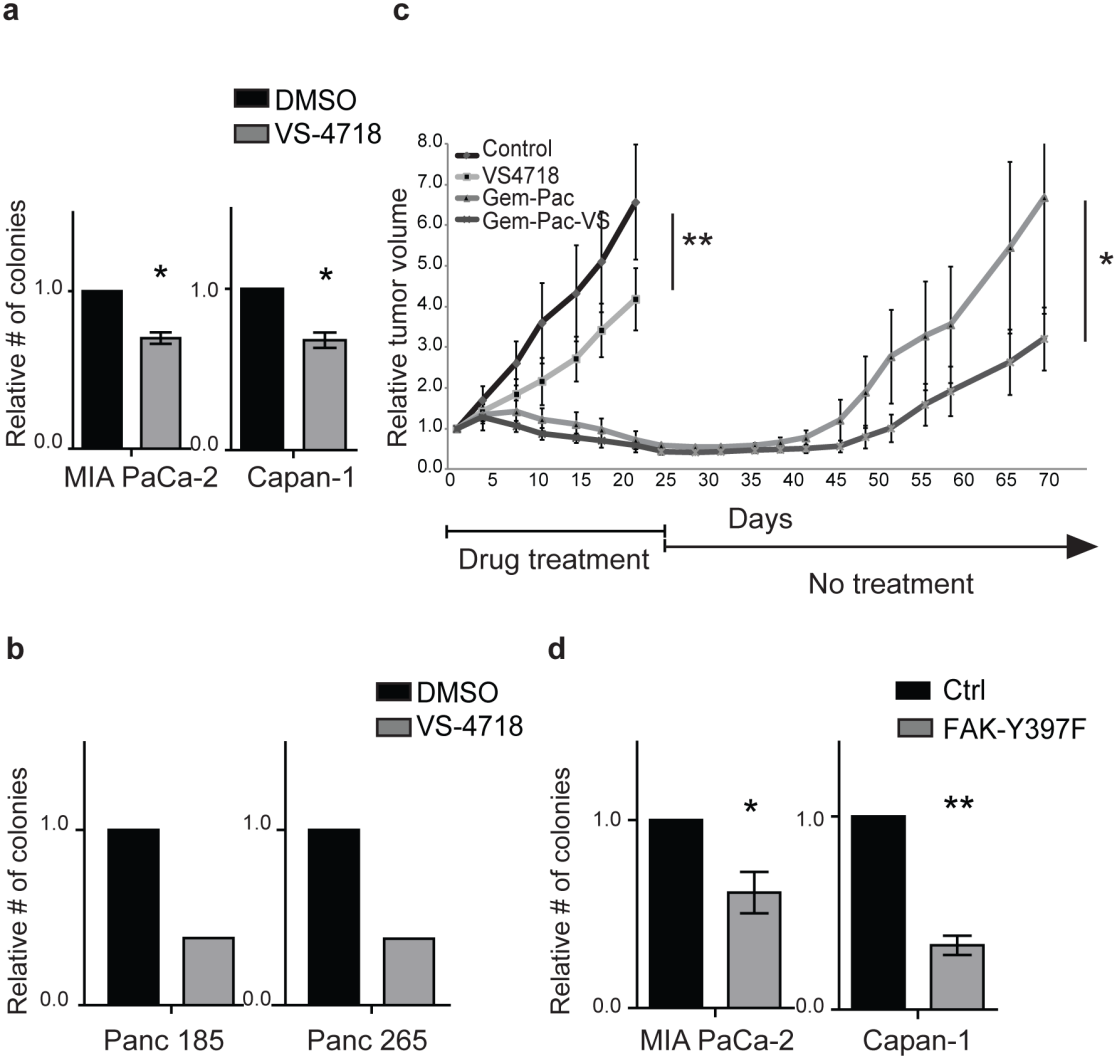
FAK kinase-inhibition decrease clonogenic PDAC growth *in vitro* and inhibits tumor growth *in vivo*. (**a**) *In vitro* colony formation by MIA PaCa-2 and Capan-1 cells following treatment with vehicle control (DMSO) or VS-4718 on type I collagen for 96 hours. Data represent mean ± SD (*n* = 3) of DMSO versus VS-4718; *P < 0.05. (**b**) Colony formation by 2 distinct patient derived xenograft cells following treatment with vehicle control (DMSO) or VS-4718 on type I collagen for 5 days. (**c**) Subcutaneous tumor growth of a patient derived xenograft (JH102) following treatment with vehicle control, VS-4718, gemcitabine plus nab-paclitaxel (Gem-Pac), or all three drugs together (Gem-Pac-VS). Five mice were included in each group. **P = 0.0076, *P = 0.03 by ANOVA. (**d**) Colony formation by MIA PaCa-2 and Capan-1 cells overexpressing FAK-Y397F following growth on type I collagen for 96 hours. Data represent mean ± SD (*n* = 4) of control versus FAK-Y397F; *P < 0.05, **P < 0.001.

FAK-FL overexpression enhanced the frequency of ALDH^+^ CSCs as well as clonogenic tumor growth and self-renewal. Therefore, FAK inhibition may serve as a strategy to target PDAC CSCs. FAK inhibitors are capable of inhibiting PDAC cell growth and survival *in vitro* [37], and several have begun clinical testing. We examined two of these agents VS-4718 and PF573228 that inhibit FAK by blocking the autophosphorylation of Y397 [48, 49] and found that both significantly reduced the clonogenic growth of PDAC cells from cell lines (Fig 4a and S4a Fig) and patient derived low-passage xenografts (Fig 4b and S4b Fig) by ≥ 3-fold. Furthermore, FAK inhibition reduced the frequency of ALDH^+^ cells in PDAC xenograft cells (S4c Fig). The inhibition of clonogenic growth was not a consequence of decreased PDAC survival as both FAK inhibitors had little effect on apoptosis (S4d Fig). We also evaluated the impact of FAK inhibition *in vivo* by treating mice bearing patient-derived PDAC xenografts with VS-4718 and/or gemcitabine plus nab-paclitaxel (Gem-Pac). Compared to vehicle-treated control animals, tumors from VS-4718 treated animals grew significantly slower (Fig 4c) and had decreased levels of pY397-FAK (S4E Fig). Similar to previous studies with gemcitabine alone [50], Gem-Pac treatment led to tumor regression, and this response was further enhanced by the addition of VS-4718 (Gem-Pac-VS-4718). After 3 weeks of treatment, tumor regrowth was significantly delayed in animals treated with Gem-Pac-VS-4718 compared to Gem-Pac alone (Fig 4c).

In addition to FAK, VS-4718 inhibits the FAK related kinase PYK2 [51]. To confirm the specificity of our pharmacologic studies, we generated cell lines overexpressing FAK in which the Y397 residue was mutated to phenylalanine (Y397F-FAK) to block its autophophorylation and activation (S4f Fig). Consistent with our drug studies, Y397F-FAK expression significantly reduced tumor colony formation by Capan-1 and MIA PaCa-2 cells cultured on type I collagen (Fig 4c). We also examined the engraftment of MIA PaCa-2 cells and found that the tumor-initiating cell (TIC) frequency of Y397F-FAK expressing cells was significantly less then control vector transfected cells (1/986 vs. 1/314; Table 1). Therefore, FAK kinase inhibition impact the tumor initiating and growth potential of PDAC cells.

### The loss of FAK inhibits PDAC self-renewal

**Fig 5:**
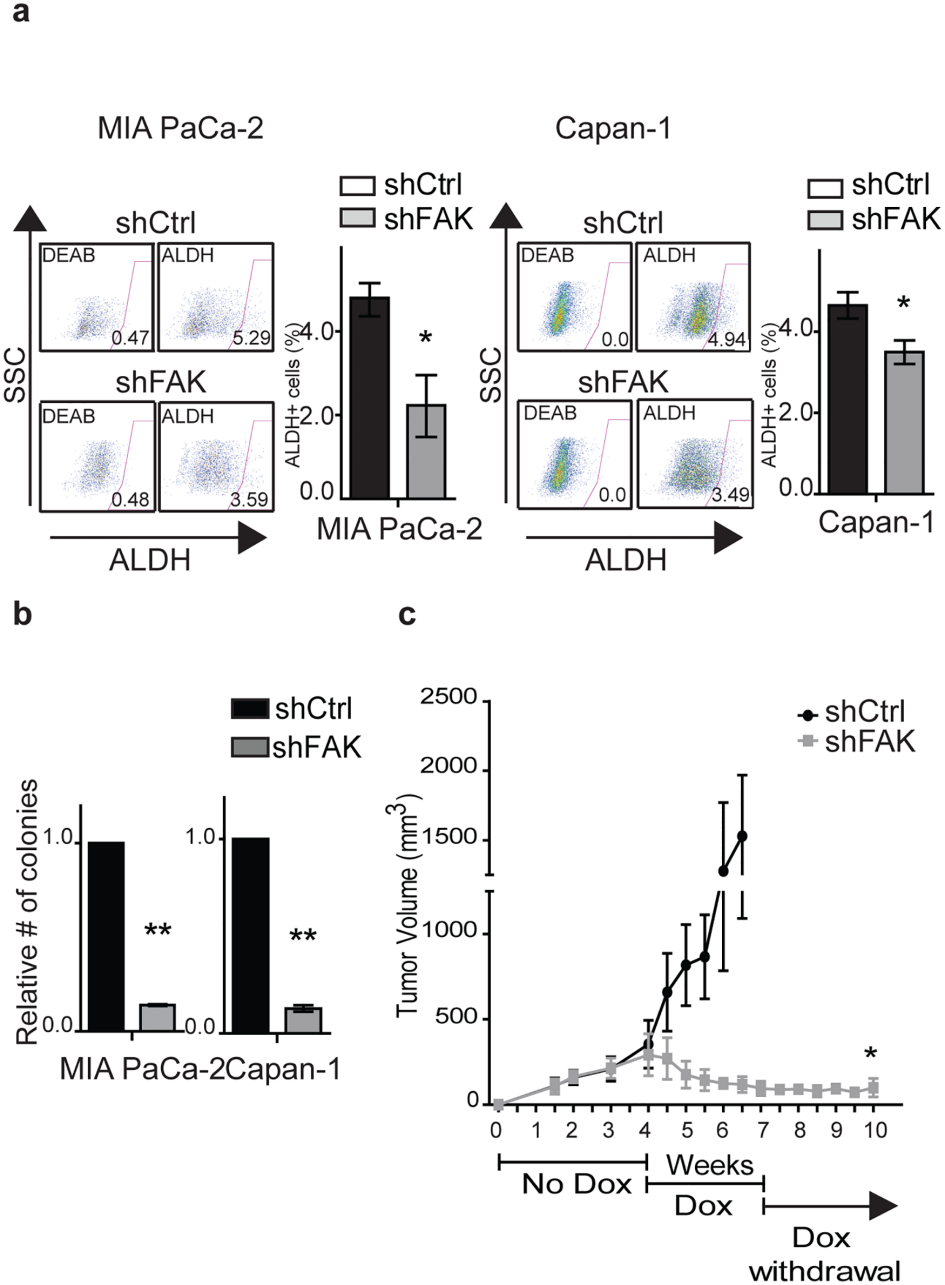
The loss of FAK inhibits self-renewal. (**a**) Frequency of ALDH^+^ MIA PaCa-2 and Capan-1 cells expressing scrambled control (shCtrl) or FAK shRNA (shFAK) following treatment with doxycycline for 96 hours. Data represent mean ± SD (*n* = 3) of shCtrl versus shFAK; *P < 0.05. (**b**) *In vitro* colony formation by MIA PaCa-2 and Capan-1 cells expressing scrambled control (shCtrl) or FAK shRNA (shFAK) following treatment with doxycycline for 96 hours on type I collagen. Data represents ± SD (*n* = 4) of control versus hairpin; **P < 0.001. (**c**) *In vivo* subcutaneous tumor growth by MIA PaCa-2 cells expressing scrambled control (shCtrl, n = 4) or FAK shRNA (shFAK, n = 7) following treatment with doxycycline. Error bar represents SD; *P = 0.02.

In addition to its kinase activity, FAK acts as an intracellular scaffold for several signaling pathways associated with tumor growth and progression [34, 52, 53], and disruption of this function can inhibit PDAC growth both *in vitro* and *in vivo* [53]. To determine whether the kinase-independent functions of FAK impact PDAC CSCs, we knocked down its expression using doxycycline-inducible shRNAs (S5a Fig). In MIA PaCa-2 and Capan-1 cells the loss of FAK expression significantly decreased the frequency of ALDH^+^ CSCs compared to cells with a control shRNA vector (Fig 5a). The loss of FAK also significantly inhibited colony formation by 72–87% compared to shRNA controls (Fig 5b) and to a greater extent than cells expressing Y397F-FAK or treated with VS-4718 (Fig 3). Similar to studies with FAK inhibitors and Y397F-FAK, the inhibition of clonogenic growth following the loss of FAK expression was not due to changes in cell proliferation or survival (S5b and S5c Fig).

We examined the impact of loss of FAK expression by examining gene expression profiles and carrying out gene set enrichment analysis (GSEA) focusing on biological pathways associated with oncogenesis. Compared to control vector MIA PaCa-2 and Capan-1 cells, FAK knock down impacted gene sets associated with CSCs (S6 Fig) [54].

To study the effects of FAK loss on *in vivo* PDAC growth, we injected shFAK-expressing cells into mice and initiated treatment with doxycycline following the development of tumors. Compared to control shRNA cells, FAK knock down led to a regression of tumors by the end of 3 weeks of treatment (Fig 5c). In contrast, the inhibition of FAK kinase activity with VS-4718 or expression of Y397F-FAK primarily slowed tumor growth compared to controls (Fig 3). Furthermore, tumor regrowth was significantly impaired following the discontinuation of doxycycline treatment and in 5 of 8 (63%) of animals, no tumors reappeared over 3 additional weeks of observation. Therefore, both the kinase and kinase-independent functions of FAK impact PDAC CSCs.

### The loss of FAK inhibits PDAC cell migration *in vitro* and metastasis *in vivo*

**Fig 6:**
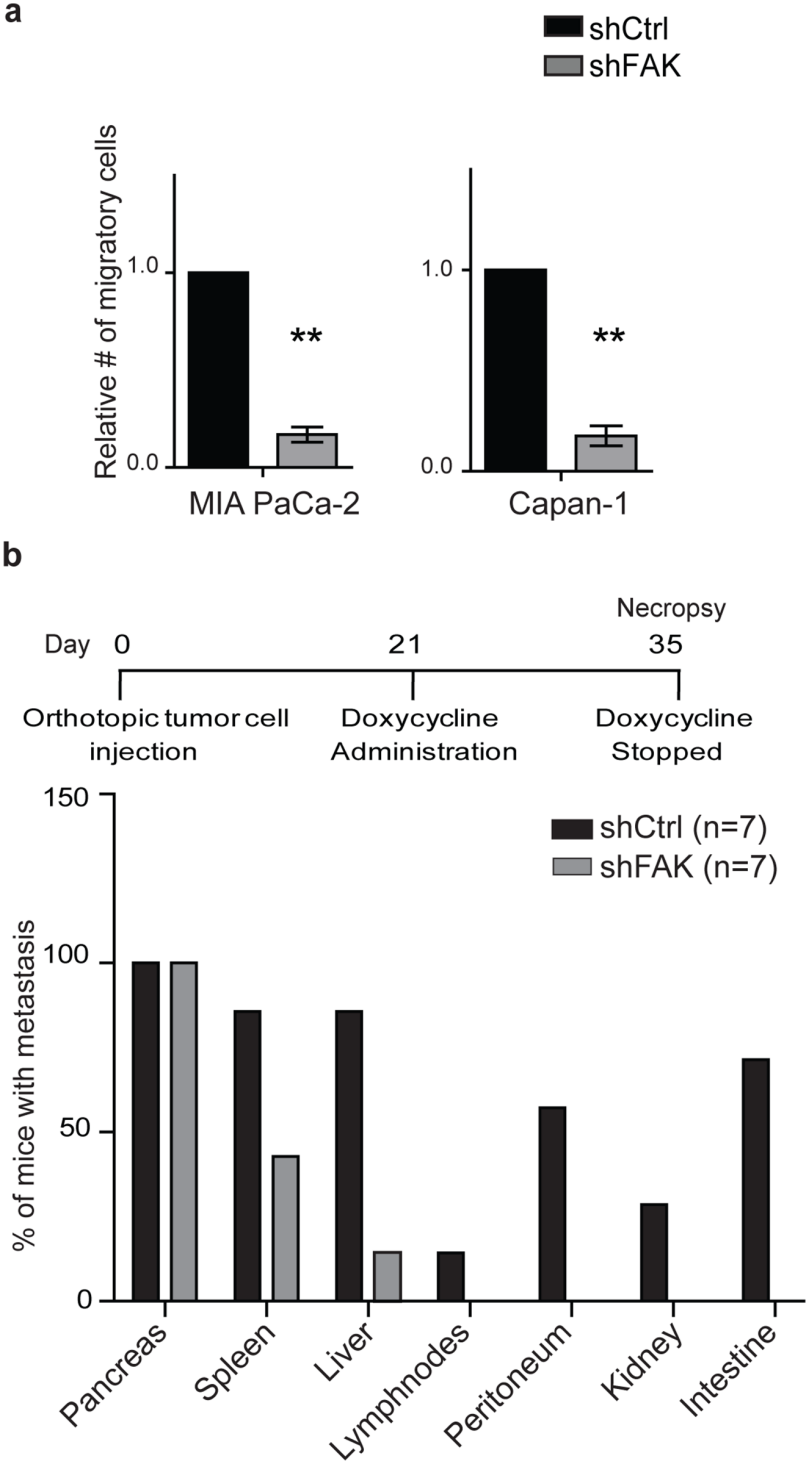
The loss of FAK inhibits PDAC cell migration *in vitro* and metastasis *in vivo*. (**a**) *In vitro* migration by MIA PaCa-2 and Capan-1 cells expressing scrambled control (shCtrl) or FAK shRNA following treatment with doxycycline on type I collagen for 96 hours. Data represent mean ± SD (*n* = 4) of control versus hairpin; **P < 0.001. (**b**) *In vivo* metastases by orthotopic MIAPaCa-2 tumors expressing scrambled control (shCtrl) or FAK shRNA (shFAK) following treatment with doxycycline. Seven mice were used in each group. Control versus hairpin; *P = 0.008.

Type I collagen and β1-integrin signaling can enhance PDAC cell migration and tissue invasion [15, 19]. Moreover, pancreatic CSCs have been implicated in PDAC dissemination and metastasis [6]. Similar to previous studies we found that type I collagen significantly enhanced *in vitro* PDAC cell migration that was dependent on β1-integrin expression (S7a and S7b Fig). FAK also regulated cell migration as treatment with VS-4718 and FAK knock down significantly decreased the migration of PDAC cells from cell lines (Fig 6a and S7c Fig).

To investigate the impact of FAK on metastasis *in vivo*, doxycycline-induced shFAK PDAC cells were orthotopically injected into the pancreata of mice and allowed to form primary tumors. Following 21 days, we treated mice with doxycycline for 14 days to knock down FAK expression, and then quantified the formation of primary tumors and metastatic lesions. There was no difference in the histological appearance of the primary pancreatic tumors derived from cells with either FAK or control shRNA vectors (S7d Fig). However, the loss of FAK expression significantly reduced the proportion of animals with metastatic lesions within the liver and spleen and prevented tumor dissemination to other visceral organs (Fig 6b). Therefore, FAK enhances the migration and metastatic potential of PDAC cells.

## Discussion

Cancer stem cells maintain long-term tumor initiating potential and promote metastatic progression in many diseases [55]. These properties suggest that the inhibition of CSCs can improve clinical outcomes, and in normal tissues and organs, stem cells are dependent on cell-extrinsic niche factors. Striking changes occur within the TME suggesting that specific extrinsic factors similarly impact CSCs. In pancreatic carcinoma, the TME is dominated by the desmoplastic reaction, and we found that type I collagen enhances tumor-initiating potential, self-renewal, and cellular migration and invasion through β1integrin and FAK signaling.

In other diseases, alterations within the TME including changes in oxygen tension, the secretion of soluble growth factors and morphogens, and the activation of non-stem cells such as cancer associated fibroblasts can regulate CSCs [56–58]. Moreover, morphologically and functionally distinct CSC niches can exist within a single tumor type and contribute to intratumoral heterogeneity. In glioblastoma, areas of hypoxia promote CSC self-renewal and drug resistance through Hypoxia Inducible Factor associated genes. Additionally, perivascular niches in which CSCs interact with non-neoplastic stellate, endothelial, and immune cells can increase self-renewal [59]. Soluble factors aberrantly secreted by non-neoplastic cells can also enhance the self-renewal of CSCs in a paracrine manner. In multiple myeloma, Growth Differentiation Factor 15 (GDF15) is secreted by bone marrow stromal cells in response to differentiated tumor cells and enhances tumor-initiating potential and self-renewal [60].

Specific soluble factors within the pancreatic TME have been found to regulate CSCs including Nodal/Activin secretion from pancreatic stellate cells, cationic antimicrobial peptide 18/LL-37 from immune cells, and IFN-stimulated factor 15 (ISG15) from tumor associated macrophages [59, 61, 62]. The desmoplastic reaction of PDAC has been thought to play a major role in therapeutic resistance by limiting drug delivery [63], and our findings demonstrate that intratumoral fibrosis also enhances tumor-initiating and self-renewal potential. Therefore, a feed forward loop may exist in PDAC in which bulk tumor cells induce cancer associated fibroblasts to secrete type I collagen that in turn expands CSCs and increase the production of differentiated cells.

FAK is pleiotropic and in addition to its kinase activity, the FERM and FAT domains provide multiple docking sites for several cellular factors including growth factor receptors, ARP2/3, Endorphilin-A2, and the E3 ubiquitin-protein ligase MDM2 (Mouse double minute 2 homolog) [64]. This scaffold function can modulate cell proliferation, survival, and the epithelial mesenchymal transition [32], and we found that loss of FAK expression by shRNA inhibited tumor growth to a greater extent than inhibiting its kinase activity and prevented tumor regrowth and metastatic dissemination. Therefore, PDAC CSCs are dependent on both kinase dependent and kinase independent functions of FAK. Although we did not identify the specific docking sites of FAK necessary for PDAC CSCs, agents capable of disrupting the scaffold function have been developed and active against breast cancer [34, 53, 65].

The effects of type I collagen on PDAC CSCs is mediated through the activation of FAK. FAK is an intracellular, multifunctional protein tyrosine kinase that interacts with integrins at the plasma membrane in response to extracellular stimuli. Structurally, it has three distinct domains: an amino-terminal FERM (band 4.1–Ezrin–Radixin–Moesin) domain; a central kinase domain; and a C-terminal focal adhesion-targeting domain [32]. Upon activation, the FAK kinase domain auto-phosphorylates the Y397 residue to recruit SRC family kinase and further phosphorylate the Y576 and Y577 residues within the catalytic domain. This canonical, kinase-dependent FAK activation is required for focal adhesion (FA) complex formation and turnover and plays an important role in cell adhesion and motility. In cancer, FAK activation promotes cancer cell survival and metastasis in a kinase dependent manner [32]. We found that the inhibition of FAK kinase activity through overexpression of FAK Y397F decreases PDAC tumor initiating potential *in vitro* and *in vivo*. Pharmacologic FAK inhibitors have been developed and demonstrated to be active against PDAC in preclinical studies [37, 40]. Furthermore, a phase I study of the FAK inhibitor VS-6062 (previously known as PF-00562271) demonstrated metabolic responses as measured by ^18^F-fluorodeoxyglucose positron emission tomography in three out of six patients with PDAC [66]. Similar to findings in ovarian cancer [67], we found that these agents decreased PDAC growth when used alone and delayed tumor regrowth in combination with cytotoxic chemotherapy. Therefore, FAK inhibitors may target PDAC CSCs especially in combination with cytotoxic chemotherapy. Based on these and other data, the FAK inhibitor VS-4718 is currently being tested in combination with gemcitabine plus nab-paclitaxel in patients in the first line pancreatic cancer setting (ClinicalTrials.gov NCT02651727).

## Materials and methods

### Cells lines and xenografts

BxPC-3, Capan-1 and MIA PaCa-2 cell lines were purchased from American Type Culture Collection and cultured on 60 mm dishes coated with or without type I collagen, fibronectin, or laminin (BD Biosciences). Prior to analysis of clonogenic growth or migration, cells were washed with 1X PBS, trypsinized, and re-suspended in medium containing 10% fetal bovine serum. Treatment with FAK-inhibitors PF573228 and VS-4718 was carried out for 72–120 hours on plastic or type I collagen.

Low passage xenografts generated from surgical specimens derived from patients undergoing surgery at the Johns Hopkins Hospital have been previously described [68, 69]. A total of 7 distinct patient-derived xenografts were studied. Single cell suspensions from patient-derived xenografts were isolated as previously described [68, 69].

### Plasmids

Full-length FAK (FAK-FL) was cloned into the NotI and BamHI sites of the pLVX-IRES-mCherry lentiviral vector (Clontech). Y397F-FAK was created using the QuikChange site-directed mutagenesis kit (Stratagene). Cell lines transduced with lentiviral constructs were selected by fluorescence activated cell sorting (FACS) based on mCherry expression. Short hairpin RNA (shRNA) constructs against FAK (shFAK-1 and shFAK-2) were cloned into the Tet-pLKO-puro lentiviral vector (Addgene). Cell lines were transduced and stable lines were selected using puromycin (Thermo Fisher Scientific).

### Animal studies

All experiments using mice were approved by the Johns Hopkins University Animal Care and Use Committee and the mice were maintained in accordance with the American Association of Laboratory Animal Care guidelines. In all studies, the appropriate measures were taken to minimize discomfort to animals. All injections and surgical procedures were performed using sterile technique with efforts made to minimize trauma to the animals. When necessary, animals were anesthetized with a mixture of 1.75% isofluorane/air. Following injections, animals were closely monitored and any that appeared moribund were immediately euthanized by administration of anesthesia, followed by inhalation of carbon dioxide until breathing ceased. Death was then ensured through cervical dislocation. Tumor growth was followed weekly by caliper measurements. For drug studies nude mice with subcutaneous low passage xenograft (JH102) were treated with VS-4718 (50mg/kg; orally; twice daily) and / or gemcitabine (25mg/kg; intraperitoneally; twice weekly) and nab-paclitaxel (50mg/kg; once weekly). Tumors were measured as previously described [68, 69]. Tumor growth comparing scrambled control or FAK shRNA expressing MIA PaCa-2 cells was carried out by administering nude mice with doxycycline (2 mg/ml) containing water, four weeks after subcutaneous injection (200,000 cells). Nude mice with orthotopic tumors were treated with doxycycline for 14 days, starting 21 days after the injection of control or FAK shRNA expressing MIA PaCa-2 cells (200,000 cells). Mice were euthanized and metastases were identified by necropsy, or in the case of the liver and spleen, hematoxylin and eosin stained histological analysis.

### Clonogenic assay

Tumor colony formation assay was carried out as previously described [68, 69]

### Quantitative real-time PCR analysis

Total RNA was extracted using the RNeasy PlusMini Kit (Qiagen) and reverse-transcribed with SuperScript III reverse transcriptase (Invitrogen). qRT-PCR was carried out using TaqMan probe against *ACTB* (Hs99999903_m1, Applied Biosystems) or *ITGB1* (Hs00559595_m1).

### Immunoprecipitation and Western bolt

Whole cell lysates (WCL) from cells were prepared using pre-chilled Mammalian Protein Extraction Reagent (Life technologies) and incubated 30 minutes on ice. The lysates were cleared by centrifugation at 13,400 rpm and 4°C for 15 min. Immunoprecipitation was carried out by incubating 500 μg of WCL with 1–2 μg of anti-FAK antibody at 4°C for 16 hours. Subsequently, 25 μg of recombinant protein A agarose beads (Thermo Scientific) were added and incubated for one hour at 4°C with slight agitation. For Western blot, proteins were separated by electrophoresis using NuPAGE^®^ Novex^®^ 4–12% Bis-Tris Protein Gels (Life technologies) and transferred to PVDF membrane. Membranes were blocked by 5% w/v BSA, 1X TBS and 0.1% Tween-20 at room temperature for one hour. Membranes were then incubated with rabbit monoclonal anti-pY397-FAK, rabbit polyclonal anti-FAK (Cell Signaling Technology), beta-tubulin, or GAPDH (Abcam) antibodies. Densitometic analyses of protein expressions were done by ImageJ software.

### Flow cytometry

Flow cytometry was carried out as previously described [68, 69]. For ALDH staining, cells from were stained with the Aldefluor reagent (Stem Cell Technologies) for 30 minutes in a 37°C water bath according to the manufacturer’s protocol. ALDH^+^ cells were identified by based on cells stained with Aldefluor in the presence of diethylaminobenzaldehyde (DEAB), a specific inhibitor of ALDH, used to control for background fluorescence. For intracellular staining, cells were fixed with 2% formalin for 10 minutes, followed by permeabilization with 1% NP40 for 10 minutes at room temperature. Cells were stained with rabbit monoclonal anti-pY397-FAK (Invitrogen), rabbit polyclonal anti-FAK (Cell Signaling), or mouse monoclonal anti-ALDH (BD Biosciences) antibodies followed by anti-rabbit-APC and anti-mouse-FITC antibodies (Invitrogen). Annexin V staining was done according to manufacturer’s protocol (eBioscience).

### *In vivo* tumor-initiating cell assay

Tumor-initiating cell (TIC) frequency was calculated by injecting 10, 100, 500, 1000 or 10,000 MIA PaCa-2 cells expressing control (vector) or FAK-FL or FAK-Y397F subcutaneously into NSG mice. TIC frequency was calculated using extreme limiting dilution analysis (ELDA) [70].

### Migration assay

Cell migration assay was carried out as previously described [68, 69]. Briefly, cells were placed in 24-well cell culture inserts containing 8-μm pores (BD Biosciences). After 72 hours, cells migrating through the filers were counted using an inverted light microscope.

### Microarray

Cells expressing inducible shRNA were treated with 50 ng/ml doxycycline for 96 hours and then RNA was isolated using RNeasy Mini Kit (Qiagen). Gene expression analysis was performed on an Illumina Human HT12_4 array at the Johns Hopkins University Sidney Kimmel Comprehensive Cancer Center Microarray Core Lab. Microarray data are available at Gene Expression Omnibus (GEO) under the accession no. GSE97488. Gene set enrichment analysis was performed with JAVA-based GSEA software [71].

### Statistics

Statistical differences between two groups were analyzed using the two-tailed, unpaired Student t test or, if described, by ANOVA (GraphPad Prism Software, Inc.).

## Supporting information

S1 Fig Type I collagen- β1 integrin signaling enhances the clonogenic growth of PDAC cells. (**a**) Relative mRNA and protein expression following knockdown of β1 integrin by shRNA in MIA PaCa-2 cells. (**b**) Primary and secondary colony formation by Capan-1 cells with scrambled control (shCtrl) or β1 integrin (shBeta1) shRNA after growth on type I collagen for 96 hours. Data represents mean ± SD (*n* = 4). **P < 0.001. (**c**) Cell viability of MIA PaCa-2 cells following knockdown of β1 integrin as assessed by annexin V staining.
(TIF)

S2 Fig Increase phopho-Y397 FAK expression by PDAC cells following overexpression of FAK-FL. Phospho-FAK (pFAK) and total FAK expression in MIA PaCa-2 and Capan-1 cells following overexpression of FAK-FL. Beta Tubulin was used as a loading control.
(TIF)

S3 Fig Type I collagen-β1-integrin-FAK signaling impacts ALDH^+^ PDAC CSCs. (**a**) α2β1 integrin expression by ALDH^+^ or all MIA PaCa-2 cells. (**b**) Phospho-FAK expression by ALDH^+^ or all MIA PaCa-2, and Capan-1 cells. (**c**) Frequency of ALDH^+^ MIA PaCa-2 cells following overexpression of FAK-FL. Data represents mean ± SD (*n* = 3). (**d**) Frequency of ALDH^+^ MIA PaCa-2 cells expressing scrambled control (shCtrl) β1 integrin (shBeta1) shRNA. Data represents mean ± SD (*n* = 3). *P < 0.05.
(TIF)

S4 Fig FAK kinase-inhibition decrease clonogenic PDAC growth *in vitro*. (**a**) *In vitro* colony formation by MIA PaCa-2 and Capan-1 cells following treatment with vehicle control (DMSO) or PF573228 on collagen I for 96 hours. Data represent mean ± SD (*n* = 4) of DMSO versus PF573228; **P < 0.001. (**b**) Colony formation by patient derived xenograft cells following treatment with vehicle control (DMSO) or PF573228 for 5 days. (**c**) Frequency of phopho-FAK^+^ (pFAK) and ALDH^+^ cells in Capan-1 and MIA PaCa-2 cell lines following treatment with DMSO or PF573228 (top panel) on type I collagen for 96 hours. Relative frequency of ALDH^+^ cells in patient derived xenografts following treatment with vehicle control (DMSO) or PF573228 (bottom panel). (**d**) Cell viability of MIA PaCa-2 cells following treatment with DMSO or VS-4718 as assessed by annexin V staining. (**e**) Ratio of pFAK to total FAK expression by JH102 xenograft cells following treatment with VS-4718, Gemcitabine plus nab-paclitaxel (Abraxane) and Gemcitabine plus nab-paclitaxel plus VS-4718. (**f**) Phospho-FAK expression in MIA PaCa-2 and Capan-1 cells overexpressing FAK-Y397F. Data represent ratio of phospho-FAK vs total FAK in percent (%).
(TIF)

S5 Fig Loss of FAK has minimal impact on PDAC cells proliferation and cell death. (**a**) FAK expression following knockdown by shRNA in MIA PaCa-2 cells. (**b**) *In vitro* cell proliferation by MIA PaCa-2 cells expressing shFAK following treatment with doxycycline for 3 days. (**c**) Cell viability of MIA PaCa-2 cells following knockdown of FAK as assessed by Annexin V staining.
(TIF)

S6 Fig FAK activation is associated with CSC gene expression signature. GSEA analysis revealed that embryonic stem cell genes signature is significantly overlapped with gene expression data set of PDAC cells expressing FAK. Control / FAK hairpin data set versus Wong cancer stem cell core data set, (NES: 2.35, *P* < 0.01 and FDR *q* = 0.01).
(TIF)

S7 Fig Type I collagen- β1 integrin-FAK signaling regulates the migration of PDAC cells. (**a**) *In vitro* migration by BxPC-3, Capan-1 and MIA PaCa-2 cells following growth on collagen I, fibronectin, or laminin for 96 hours. Data represent mean ± SD (*n* = 4) compared to control; *P < 0.05; **P < 0.001. (**b**) Migration by MIA PaCa-2 and Capan-1 cells expressing shBeta1 following growth on collagen I for 96 hours. Data represent mean ± SD (*n* = 4) of control versus shBeta1; *P < 0.05. (**c**) *In vitro* migration by MIA PaCa-2 cells following treatment with DMSO or VS-4718 on collagen I for 96 hours. Data represent mean ± SD (*n* = 4) of DMSO versus VS-4718; *P < 0.05. (d) Hematoxylin and eosin (H&E) staining of orthotopically grown MIA PaCa-2 tumors and metastases.
(TIF)
