# Supplementary figures and images for: The extracellular matrix and focal adhesion kinase signaling regulate cancer stem cell function in pancreatic ductal adenocarcinoma
Source: PLoS One. 2017 Jul 10;12(7):e0180181. doi: 10.1371/journal.pone.0180181 (PMC5503247; doi:10.1371/journal.pone.0180181)

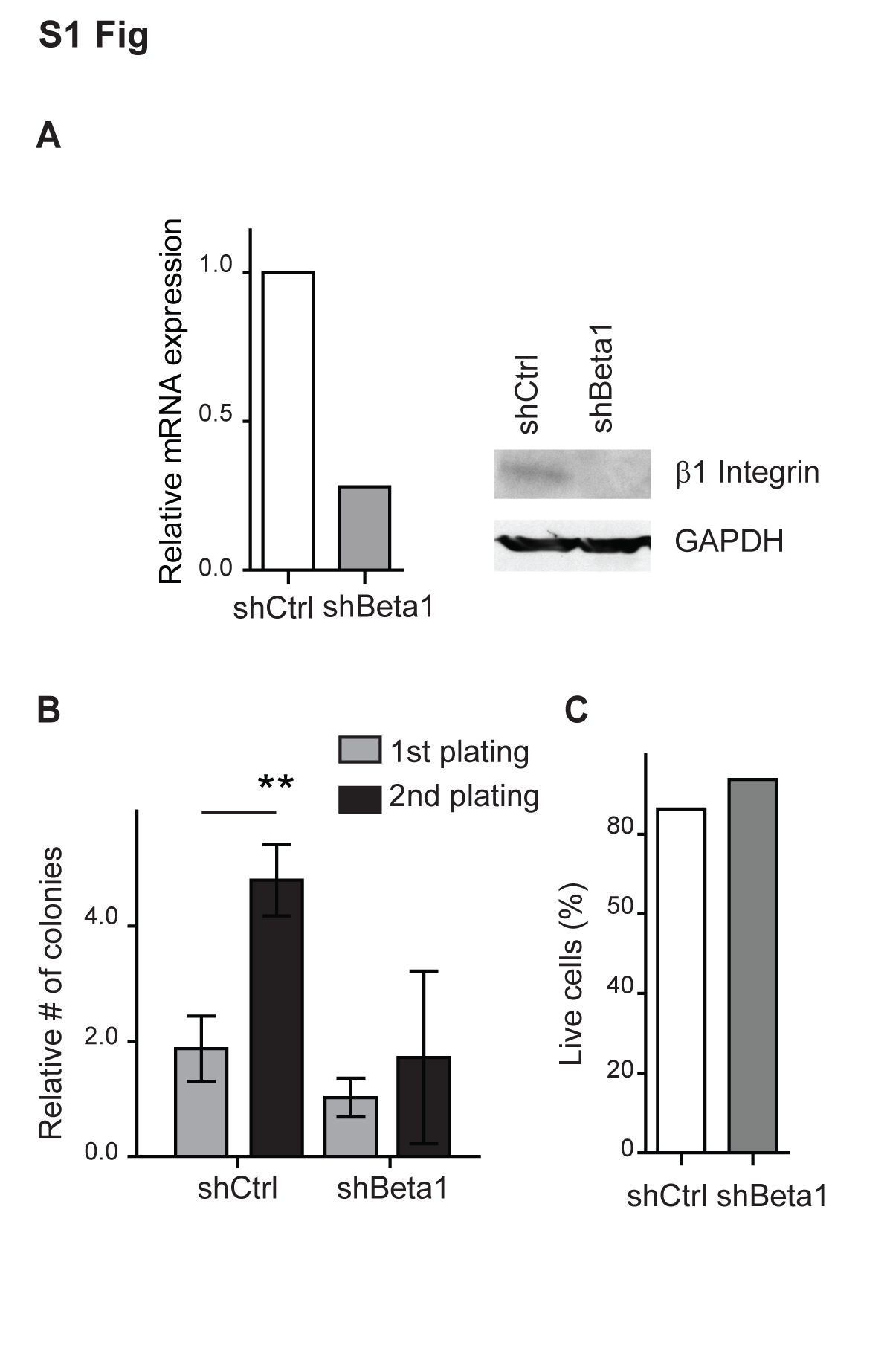

Supplement: S1 Fig — (a) Relative mRNA and protein expression following knockdown of β1 integrin by shRNA in MIA PaCa-2 cells. (b) Primary and secondary colony formation by Capan-1 cells with scrambled control (shCtrl) or β1 integrin (shBeta1) shRNA after growth on type I collagen for 96 hours. Data represents mean ± SD (n = 4). **P < 0.001. (c) Cell viability of MIA PaCa-2 cells following knockdown of β1 integrin as assessed by annexin V staining. (TIF) [file pone.0180181.s001.tif]

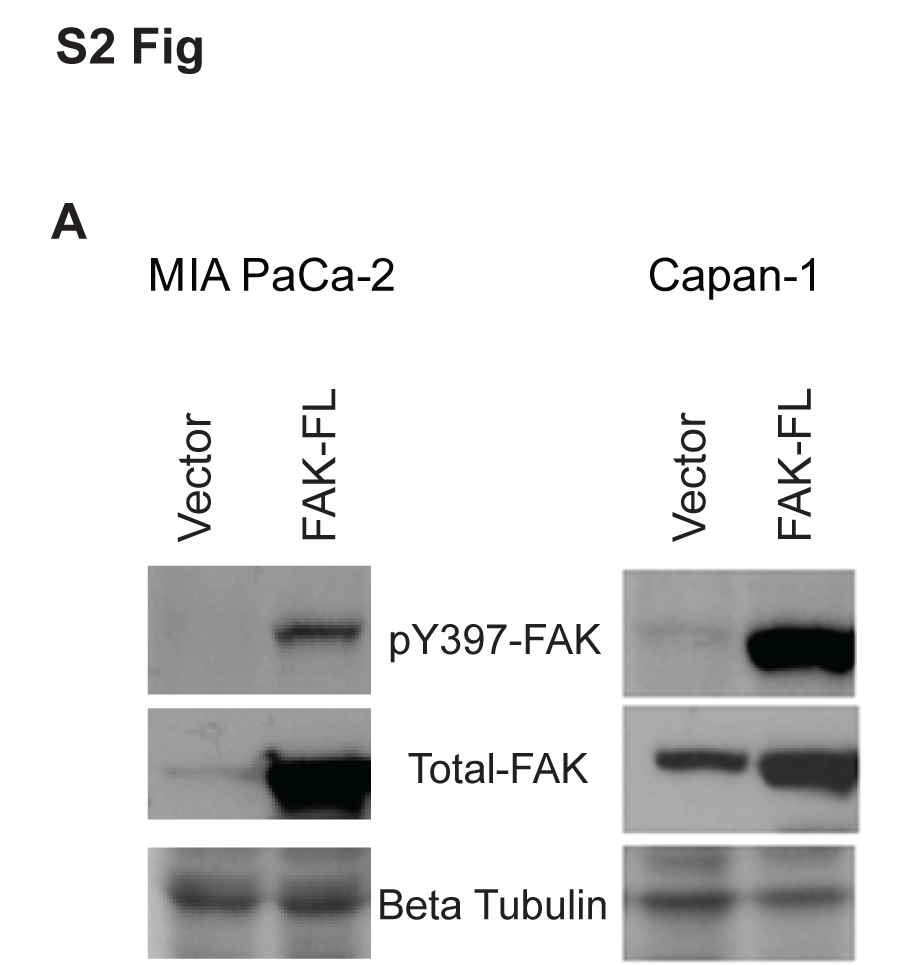

Supplement: S2 Fig — Phospho-FAK (pFAK) and total FAK expression in MIA PaCa-2 and Capan-1 cells following overexpression of FAK-FL. Beta Tubulin was used as a loading control. (TIF) [file pone.0180181.s002.tif]

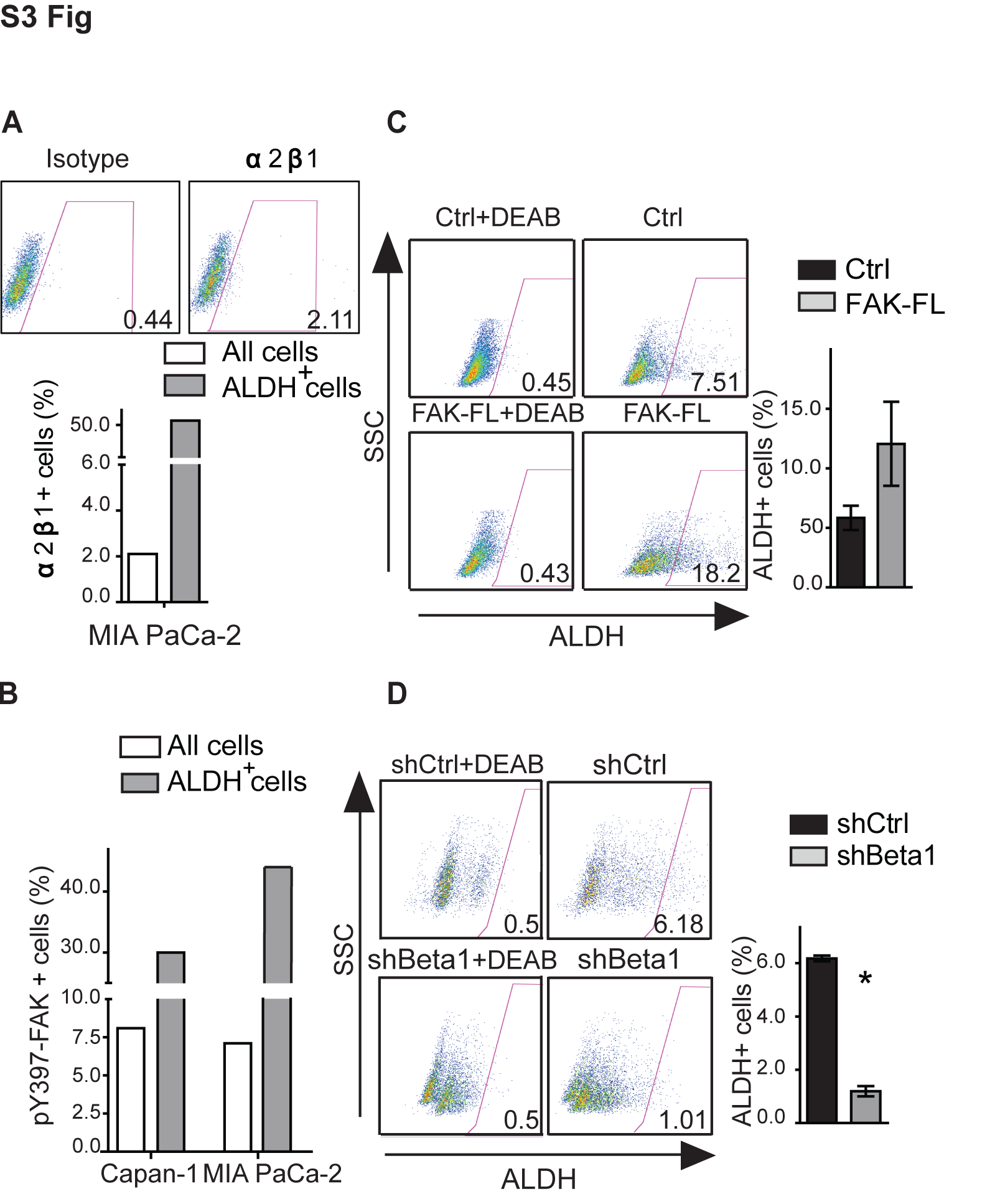

Supplement: S3 Fig — (a) α2β1 integrin expression by ALDH+ or all MIA PaCa-2 cells. (b) Phospho-FAK expression by ALDH+ or all MIA PaCa-2, and Capan-1 cells. (c) Frequency of ALDH+ MIA PaCa-2 cells following overexpression of FAK-FL. Data represents mean ± SD (n = 3). (d) Frequency of ALDH+ MIA PaCa-2 cells expressing scrambled control (shCtrl) β1 integrin (shBeta1) shRNA. Data represents mean ± SD (n = 3). *P < 0.05. (TIF) [file pone.0180181.s003.tif]

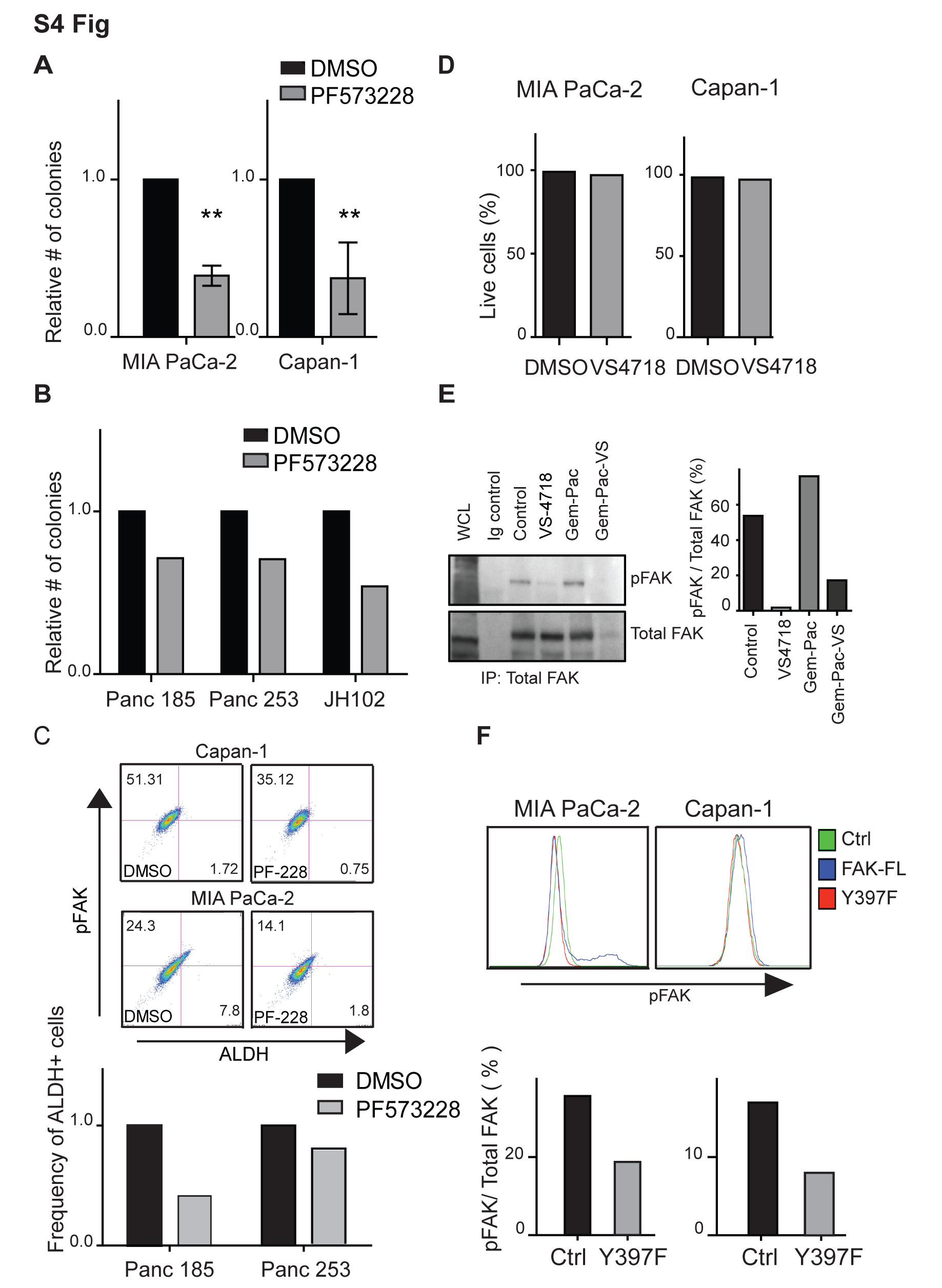

Supplement: S4 Fig — (a) In vitro colony formation by MIA PaCa-2 and Capan-1 cells following treatment with vehicle control (DMSO) or PF573228 on collagen I for 96 hours. Data represent mean ± SD (n = 4) of DMSO versus PF573228; **P < 0.001. (b) Colony formation by patient derived xenograft cells following treatment with vehicle control (DMSO) or PF573228 for 5 days. (c) Frequency of phopho-FAK+ (pFAK) and ALDH+ cells in Capan-1 and MIA PaCa-2 cell lines following treatment with DMSO or PF573228 (top panel) on type I collagen for 96 hours. Relative frequency of ALDH+ cells in patient derived xenografts following treatment with vehicle control (DMSO) or PF573228 (bottom panel). (d) Cell viability of MIA PaCa-2 cells following treatment with DMSO or VS-4718 as assessed by annexin V staining. (e) Ratio of pFAK to total FAK expression by JH102 xenograft cells following treatment with VS-4718, Gemcitabine plus nab-paclitaxel (Abraxane) and Gemcitabine plus nab-paclitaxel plus VS-4718. (f) Phospho-FAK expression in MIA PaCa-2 and Capan-1 cells overexpressing FAK-Y397F. Data represent ratio of phospho-FAK vs total FAK in percent (%). (TIF) [file pone.0180181.s004.tif]

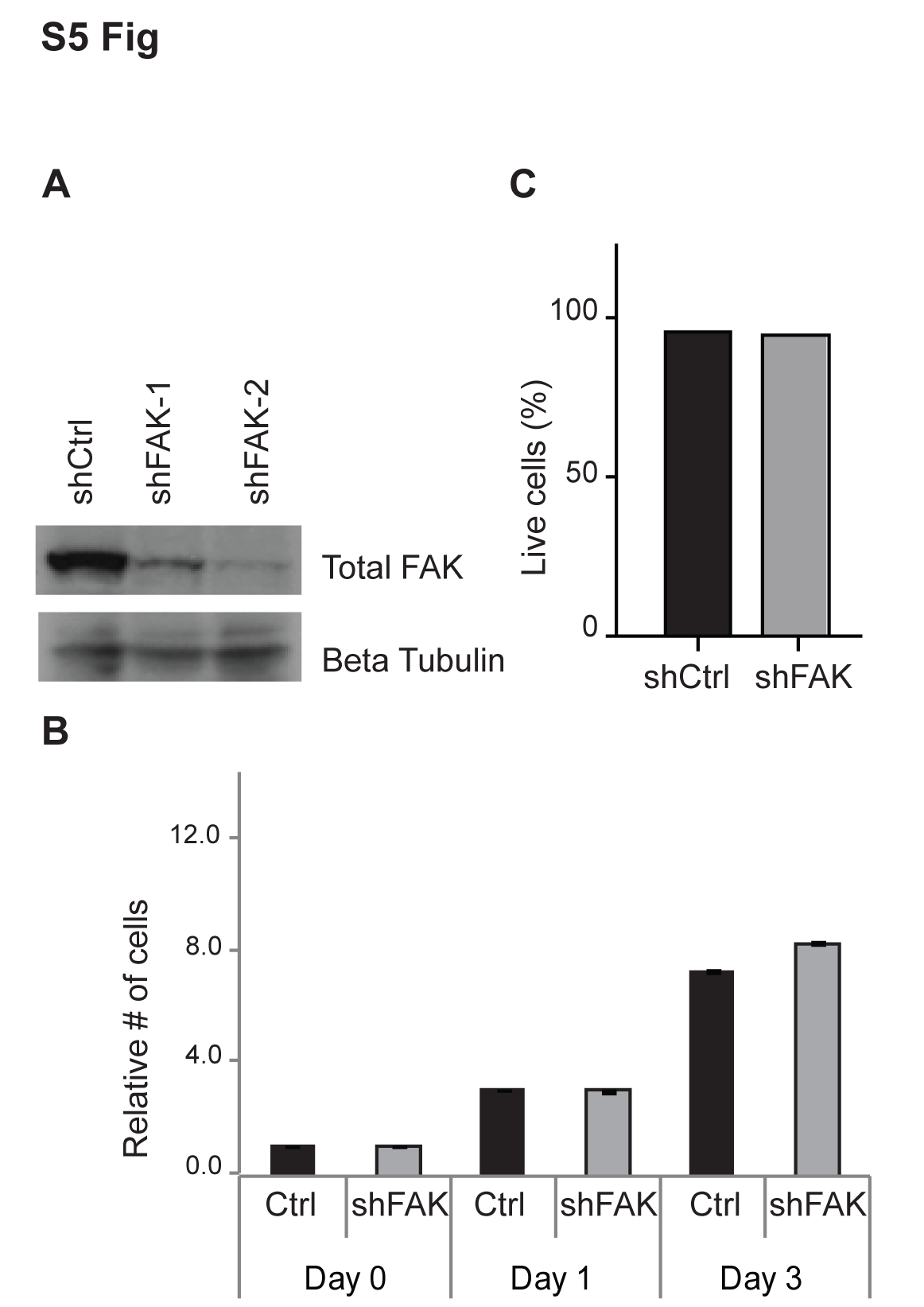

Supplement: S5 Fig — (a) FAK expression following knockdown by shRNA in MIA PaCa-2 cells. (b) In vitro cell proliferation by MIA PaCa-2 cells expressing shFAK following treatment with doxycycline for 3 days. (c) Cell viability of MIA PaCa-2 cells following knockdown of FAK as assessed by Annexin V staining. (TIF) [file pone.0180181.s005.tif]

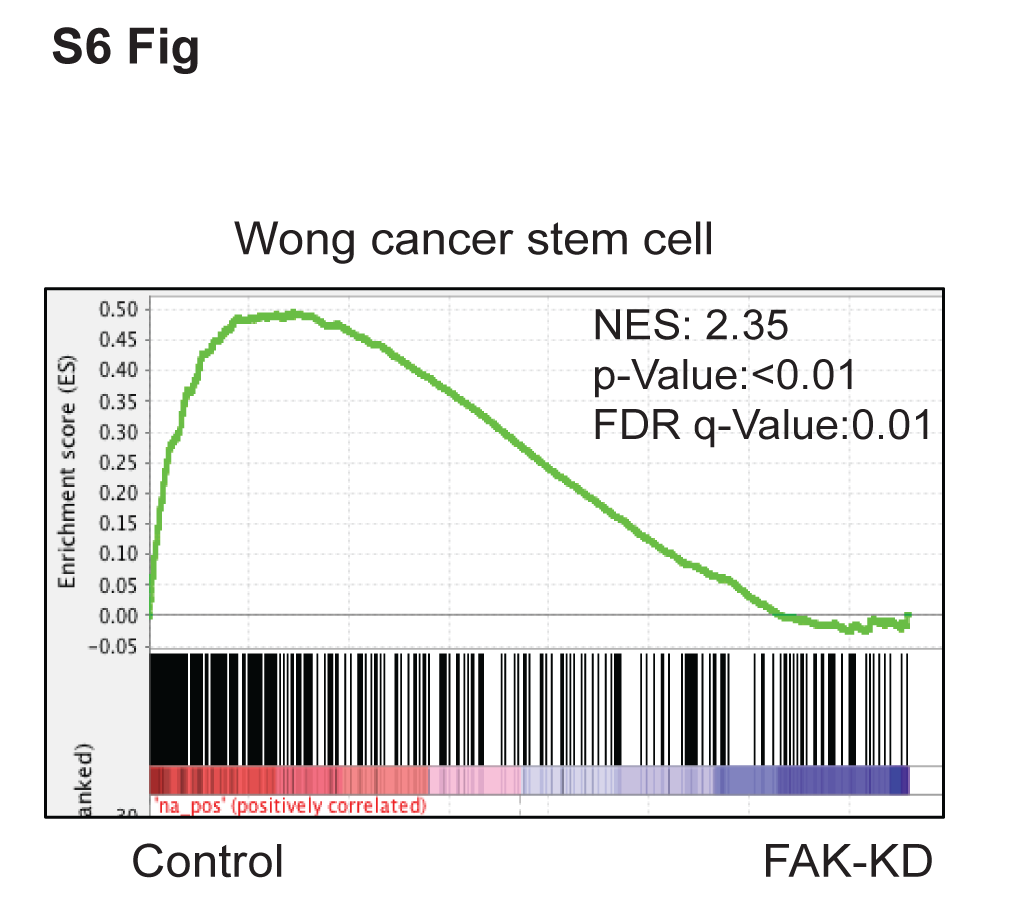

Supplement: S6 Fig — GSEA analysis revealed that embryonic stem cell genes signature is significantly overlapped with gene expression data set of PDAC cells expressing FAK. Control / FAK hairpin data set versus Wong cancer stem cell core data set, (NES: 2.35, P < 0.01 and FDR q = 0.01). (TIF) [file pone.0180181.s006.tif]

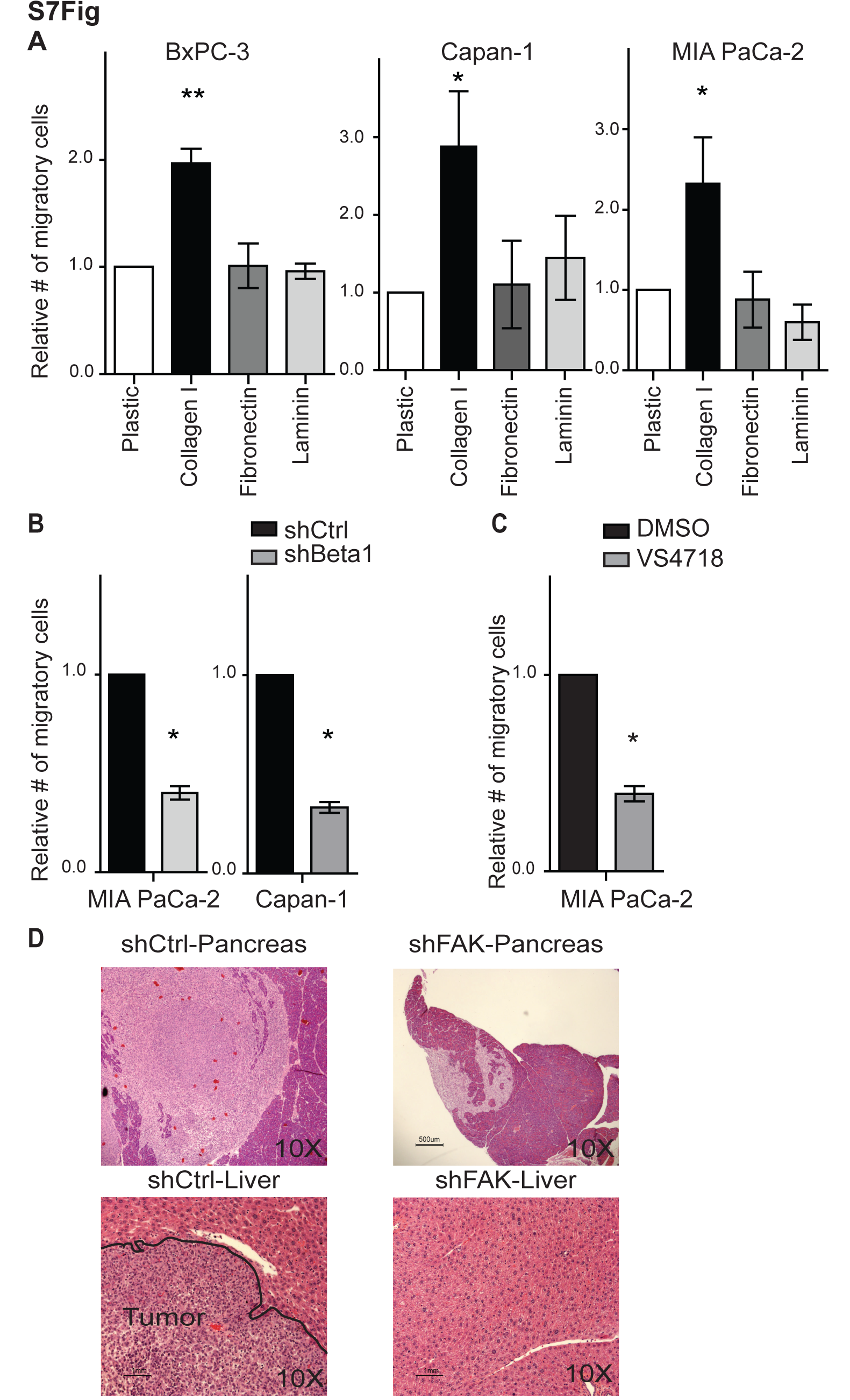

Supplement: S7 Fig — (a) In vitro migration by BxPC-3, Capan-1 and MIA PaCa-2 cells following growth on collagen I, fibronectin, or laminin for 96 hours. Data represent mean ± SD (n = 4) compared to control; *P < 0.05; **P < 0.001. (b) Migration by MIA PaCa-2 and Capan-1 cells expressing shBeta1 following growth on collagen I for 96 hours. Data represent mean ± SD (n = 4) of control versus shBeta1; *P < 0.05. (c) In vitro migration by MIA PaCa-2 cells following treatment with DMSO or VS-4718 on collagen I for 96 hours. Data represent mean ± SD (n = 4) of DMSO versus VS-4718; *P < 0.05. (d) Hematoxylin and eosin (H&E) staining of orthotopically grown MIA PaCa-2 tumors and metastases. (TIF) [file pone.0180181.s007.tif]
